# Supplementary material for: A Systematic Review of Isotopically Measured Iron Absorption in Infants and Children Under 2 Years
Source: Nutrients. 2024 Nov 8;16(22):3834. doi: 10.3390/nu16223834 (PMC11597703; doi:10.3390/nu16223834)
Supplement: Supplementary file 1 [file nutrients-16-03834-s001.zip › nutrients-3255585-supplementary.pdf]

Supplemental File

Section S1: Search Strategy

A. PubMed

Words included as [tiab] or MeSH terms as these terms are expected to come up in Title/Abstract (or be classified according to corresponding MeSH terms).

(((Iron[tiab] OR "Iron"[Mesh]) AND (absorb[tiab] OR absorption[tiab] OR "Absorption"[Mesh] OR absorptive[tiab])) AND ("Hepcidins"[Mesh] OR Hepcidin[tiab] OR Prohepcidin[tiab] OR "Liver Expressed Antimicrobial Peptide"[tiab] OR "Liver-Expressed Antimicrobial Peptide"[tiab] OR "isotope label"[tiab] OR "isotopic label"[tiab] OR "zinc protoporphyrin"[tiab] OR ZnPP[tiab] OR hematocrit[tiab] OR hemoglobin[tiab] OR ferritin[tiab])) AND ("Infant"[Mesh] OR Infant[tiab] OR "Child"[Mesh] OR Child[tiab] OR children[tiab] OR baby[tiab] OR newborn[tiab] OR postnatal[tiab] OR Pre-term[tiab] OR "Pre term"[tiab] OR post-natal[tiab])

B. EMBASE

('iron'/exp OR iron:ti,ab) AND ('absorption'/exp OR absorption:ti,ab OR absorb:ti,ab OR absorptive:ti,ab) AND ('infant'/exp OR 'child'/exp OR infant:ti,ab OR baby:ti,ab OR newborn:ti,ab OR postnatal:ti,ab OR 'pre term':ti,ab OR 'post natal':ti,ab OR child:ti,ab OR children:ti,ab) AND ('hepcidin'/exp OR 'liver expressed antimicrobial peptide':ti,ab OR 'liver-expressed antimicrobial peptide':ti,ab OR ‘isotope labeling’/exp OR ‘protoporphyrin zinc’/exp OR ‘hematocrit’/exp OR ‘hemoglobin’/exp OR ‘ferritin’/exp) AND [humans]/lim

C. SCOPUS

ABS ((((iron) AND (absorb OR absorption OR absorptive)) AND (hepcidin OR prohepcidin OR "Liver Expressed Antimicrobial Peptide" OR "Liver-Expressed Antimicrobial Peptide" OR "isotope label" OR isotopic AND label OR "zinc protoporphyrin" OR znpp OR hematocrit OR hemoglobin OR ferritin)) AND (infant OR child OR children OR baby OR newborn OR postnatal OR pre-term OR "Pre term" OR post-natal))

D. Web of Science

(AB=(iron)) AND (AB=(absorption)) AND ((AB=(hepcidin)) OR (AB=(prohepcidin)) OR (AB=(Liver Expressed Antimicrobial Peptide)) OR (AB=(Liver-Expressed Antimicrobial Peptide)) OR (AB=(isotope label)) OR (AB=(isotopic label)) OR (AB=(zinc protoporphyrin)) OR (AB=(ZnPP)) OR (AB=(hematocrit)) OR (AB=(hemoglobin)) OR (AB=(ferritin))) AND ((AB=(Infant)) OR (AB=(Child)) OR (AB=(children)) OR (AB=(baby)) OR (AB=(newborn)) OR (AB=(postnatal)) OR (AB=(pre-term)) OR (AB=(pre term)) OR (AB=(post-natal)))

Section S2: Risk of Bias Assessment

Supplemental Figure S1: Risk of Bias Summary. Review authors' judgments about each risk of bias item for each included study

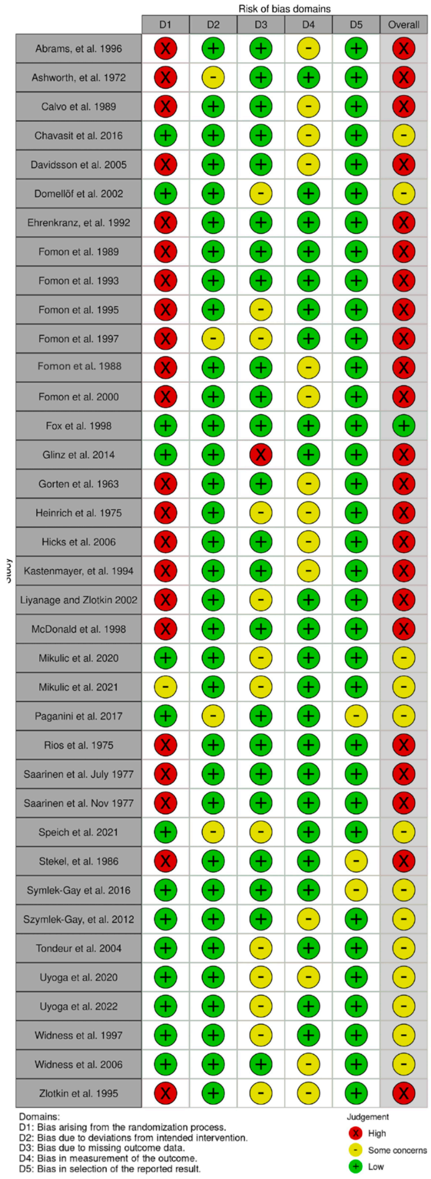

Supplemental Figure S2: Risk of Bias Graph. review authors' judgments about each risk of bias item presented as percentages across all included studies.

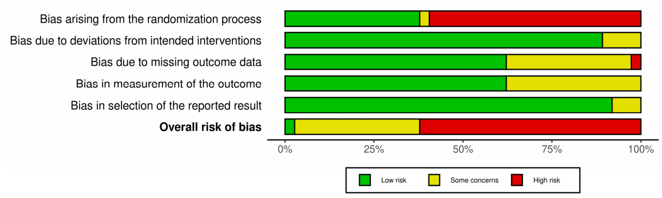

<u>Supplemental Table S1</u>: Iron Absorption and Red Blood Cell Incorporation

References	Population characteristics	Interventions	Outcomes
Gorten et al. (1963) [35]	• 14 preterm infants born at 978-2112 g 1-10 wks of age	• Formula w/ or w/out $FeSO_4$ prior to testing<br>• Test feeding $^{59}FeCl_3$ in formula given at 758 μg vs. 491 μg of iron	• Iron absorption rate (31.5%) correlated w/ rate of growth<br>• Iron absorption rate is higher in lower vs higher test dose (40.9% vs 18.9%)<br>• Erythrocyte incorporation rate (15.3%) correlated w/ rate of growth and erythropoiesis
Ehrenkranz et.al. (1992) [26]	• 11 preterm infants born at 24-33 wks and 780-1520 g	• $^{58}FeSO_4$ 228 μg/kg Fe plus 10 mg/kg vitamin C	▪ Iron absorption was 41.6 ± 17.6%<br>▪ Iron red blood cell incorporation was 12 ± 9.6% of the ^{58}Fe dose
Kastenmayer et.al. (1994) [38]	• 9 infants 13–25 wks old	• 2.5 mg ^{57}Fe or 0.6 mg ^{58}Fe plus 1.9 mg normal Fe in formula	• No difference in absorption of the two isotopes
Zlotkin et.al. (1995) [56]	• 6 preterm infants born <1500 g<br>• Mean gestational age 27 wks<br>• Mean postnatal age 4 wks	• Intravenous 15 mg/kg $^{57}FeSO_4$<br>• Enteral iron 1.5 mg/kg $^{58}FeSO_4$	• Erythrocyte incorporation of iron was 17.8% of intravenous dose and 4.4% of enteral dose
Fomon et.al. (1988) [27]	• 9 infants 126-day old	• ^{58}Fe	• Erythrocyte incorporation inversely correlated with serum ferritin concentration
McDonald et.al. (1998) [40]	• 13 preterm infants born at 27-30 wks and <1500 g 1-4 wks of age	• Fed formula with 2.2 mg/kg/d of iron prior to testing<br>• D1: 0.7 mg of ^{57}Fe with low iron formula<br>• D2: 2.0 mg ^{54}Fe with multivitamin supplement<br>• D14: 0.2 mg of ^{58}Fe intravenous	• Red blood cell incorporation of iron from supplement was greater than from premature formula<br>• Red blood cell incorporation rate correlated with reticulocyte count but not Hb
Fomon et.al. (2000) [32]	• Study 1: 7 infants at 54-165 days old<br>• Study 2: 18 infants at 20-215 days old	• ^{58}Fe for 14 days (study 1) or 11 days (study 2)	• Fecal excretion of ingested ^{58}Fe predominantly during first 4 days but continues beyond 7 days

	• *1 infant in study 2 also participated in study 1		• Far less than 80% of retained isotope is incorporated into erythrocytes
Szymlek-Gay et.al. (2016) [50]	• 72 iron sufficient infants 6 mos old	• FeSO4 6.6 mg/d in formula, 1.3 mg/d in formula or 6.6 mg/d in drops for 45 days<br>• Test ^{57}Fe and ^{58}Fe given on study day 31 (at 7 mos of age).	• No differences in iron absorption and erythrocyte incorporation
Speich et.al. (2021) [47]	• 22 infants 14–20 mos old	• 12 mg/d $^{57}FeSO_4$ followed by no iron	• Iron absorption 3-8x higher and iron loss 3-4x higher during iron supplementation than during un-supplemented period.

$FeSO_4$ = Iron sulphate, ^{57}Fe = stable isotope iron 57, ^{58}Fe = stable isotope iron 58, ^{59}Fe = radioisotope iron 59, mos = months, wks = weeks

Supplemental Table S2: Iron Absorption With Different Iron Forms and Complementary Foods

References	Population Characteristics	Interventions	Outcomes
Ashworth et al. (1973) [21]	42 infants<br>5– 24 mos old	The groups: ^{59}Fe in maize, baked soybeans, boiled soybeans, ferrous ascorbate	▪ Iron absorption from maize was low
Rios et al. (1975) [44]	67 infants<br>4–7 mos old	Sodium iron pyrophosphate and ferric orthophosphate in cereal or $^{55}FeSO_4$ in formulas	▪ Sodium iron pyrophosphate and ferric orthophosphate in cereal were poorly absorbed<br>▪ Small particles of reduced iron and $FeSO_4$ in cereal were absorbed better
Calvo et al. (1989) [22]	10 infants<br>8–10 mos old	Bovine hemoglobin fortified cereal or ferrous ascorbate	▪ Mean iron absorption of hemoglobin-cereal was lower compared to ferrous ascorbate
Fomon et al. (1989) [28]	49 infants<br>3–4 mos old	▪ $FeSO_4$ in cereal-fruit or enriched rice cereal or vegetable-beef or grape juice<br>▪ $FeSO_4$ or ferrous fumarate in enriched rice cereal	▪ Erythrocyte incorporation was low from enriched rice cereal fortified w/ $FeSO_4$ and vegetable-beef meals

			▪ Other test meals provided about 20-26% of daily iron requirement
Fox et al. (1998) [33]	46 infants (n=22 for study 1 and n=24 for study 2)<br>9 mos old	▪ <u>Study 1</u>: $^{57/58}Fe$ glycine vs. $FeSO_4$<br>▪ <u>Study 2</u>: $^{57/58}Fe$ glycine or $FeSO_4$ with foods high in phytate	▪ No differences in bioavailability between iron glycine and $FeSO_4$<br>▪ Both iron compounds had lower bioavailability from high-phytate food
Liyanage and Zlotkin (2002) [39]	39 infants<br>7-12 mos old	Encapsulated or non-encapsulated ferrous fumarate sprinkles in rice or wheat-based meals	▪ Absorption from non-encapsulated iron was higher<br>▪ No effect of type of cereal on iron absorption
Davidsson et al. (2005) [24]	11 infants<br>4–7 mos old	NaFeEDTA or $^{58}FeSO_4$ plus ascorbic acid in wheat or soy-based baby foods	▪ No difference in iron bioavailability between the iron groups and test meals
Chavasit et al. (2015) [23]	30 infants<br>8–24 mos old	Ferric ammonium citrate or $FeSO_4$ + NaFeEDTA in rice	▪ Iron absorption of FeSO4+NaFeEDTA was higher than ferric ammonium citrate
Mikulic et.al. (2021) [42]	23 infants<br>6–14 mos old<br>87% iron deficient<br>70% anemic	▪ ^{57}Fe fumarate + $Na^{58}FeEDTA$ or $^{54}FeSO_4$ w/ and w/out GOS<br>▪ All infants received all 4 test meals in maize porridge randomly on days 2, 3, 19, and 20.	▪ Iron absorption from $FeSO_4$ was higher than from Fe fumarate + NaFeEDTA w/ and w/out GOS<br>▪ GOS had no significant effect on iron absorption
Uyoga et al. (2022) [53]	30 infants<br>6–14 mos old	▪ $^{54/57}Fe$ fumarate in refined wheat flour or different whole grain cereals<br>▪ $^{54/57}Fe$ fumarate or ^{58}Fe bisglycinate in whole grain oat-based cereals	▪ Meal predicted of fractional iron absorption<br>▪ Whole grain oat cereal had lower iron absorption

^{57}Fe = stable isotope iron 57, ^{58}Fe = stable isotope iron 58, ^{59}Fe = radioisotope iron 59, mos = months, wks = weeks

<u>Supplemental Table S3:</u> Summary of Iron Absorption Main Findings

Factors	Consistency Among Studies	References
Breast milk has high bioavailability	Consistent	45, 29, 30,
Higher iron content in formula (>4-12 mg/L) doesn't improve iron status	Consistent	46, 31, 50
Ascorbic acid (Vitamin C) increases iron absorption	Consistent	48, 20
Exogenous erythropoietin doesn't improve iron absorption	Consistent	54, 55
Unmodified cow's milk reduces iron absorption	Consistent	36, 46
Lactoferrin facilitates iron absorption	Insufficient	41
Probiotics facilitates iron absorption	Not consistent	43, 42,
Optimal iron dose, frequency, form, and complimentary foods	Not consistent	25, 34, 52, 51, 50,
